# Gold(III) Porphyrin Was Used as an Electron Acceptor for Efficient Organic Solar Cells

**DOI:** 10.1021/acsami.1c22813

**Published:** 2022-02-23

**Authors:** Virginia Cuesta, Manish Kumar Singh, Edgar Gutierrez-Fernandez, Jaime Martín, Rocío Domínguez, Pilar de la Cruz, Ganesh D. Sharma, Fernando Langa

**Affiliations:** † Institute of Nanoscience, Nanotechnology and Molecular Materials (INAMOL), Universidad de Castilla-La Mancha, Campus de la Fábrica de Armas, Toledo 45071, Spain; ‡ Department of Physics, The LNM Institute of Information Technology (Deemed University), Jamdoli, Jaipur (Raj.) 302031, India; § POLYMAT, University of the Basque Country, UPV/EHU Av. de Tolosa 72, San Sebastián 20018, Spain; ∥ Ikerbasque Basque Foundation for Science, Bilbao 48013, Spain; ⊥ Universidade da Coruña, Grupo de Polímeros, Centro de Investigacións Tecnolóxicas (CIT), Esteiro, Ferrol 15471, Spain

**Keywords:** gold(III) porphyrin, nonfullerene acceptor, organic photovoltaics, bulk heterojunction, near-infrared absorption

## Abstract

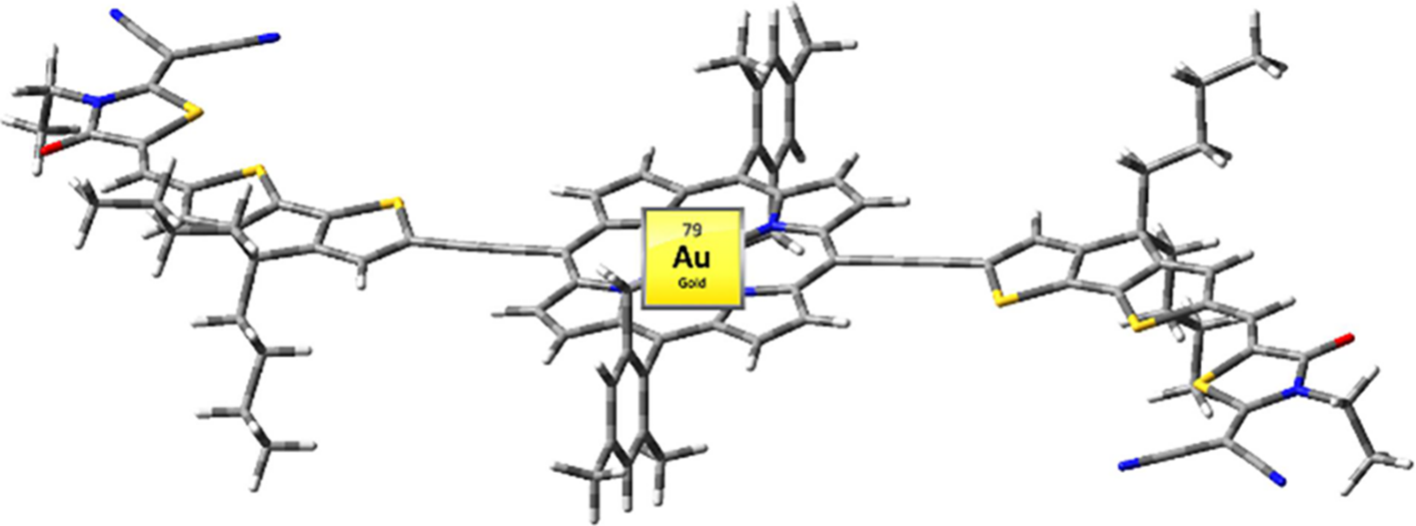

The widespread use of nonfullerene-based electron-accepting materials has triggered a rapid increase in the performance of organic photovoltaic devices. However, the number of efficient acceptor compounds available is rather limited, which hinders the discovery of new, high-performing donor:acceptor combinations. Here, we present a new, efficient electron-accepting compound based on a hitherto unexplored family of well-known molecules: gold porphyrins. The electronic properties of our electron-accepting gold porphyrin, named **VC10**, were studied by UV–Vis spectroscopy and by cyclic voltammetry (CV) , revealing two intense optical absorption bands at 500–600 and 700–920 nm and an optical bandgap of 1.39 eV. Blending **VC10** with PTB7-Th, a donor polymer, which gives rise to an absorption band at 550–780 nm complementary to that of **VC10**, enables the fabrication of organic solar cells (OSCs) featuring a power conversion efficiency of 9.24% and an energy loss of 0.52 eV. Hence, this work establishes a new approach in the search for efficient acceptor molecules for solar cells and new guidelines for future photovoltaic material design.

## Introduction

Organic solar cells (OSCs) based on solution-processed bulk heterojunction (BHJ) active layers have emerged as promising solutions for the conversion of solar energy into electrical energy in building and indoor applications due to their unique advantages, such as being lightweight and semitransparent and the possibility of being processed by low-cost roll-to-roll methods.^1−6^ A blend of an electron-donating material and an electron-accepting material forms the BHJ active layer, creating internal donor–acceptor heterojunctions, and their optical and electrochemical properties are very important for the realization of a high power conversion efficiency (PCE).^7−9^

In the early stage of OSCs, fullerene and its derivatives (e.g., PC_61_BM and PC_71_BM) have been extensively employed as electron acceptors in OSCs due to their high electron mobility and isotropic charge transfer in the BHJ active layer.^10−14^ However, the PCEs of fullerene-based OSCs have been restrained to approximately 11% due to their low absorption coefficients in the visible region of the solar spectrum, their high cost of synthesis and purification, and the difficulty in tuning their frontier energy levels.^15−18^

In recent years, nonfullerene small-molecule acceptors (NFSMAs) have attracted extensive interest due to their promising characteristics, such as easy approachability, better absorption covering both visible and near-infrared (NIR) regions, and tunable frontier energy levels,^19−22^ and the PCE of OSCs has reached values in the range of 17–18% for a single junction active layer.^23−27^ It was predicted that a PCE greater than 20% can be attained by employing suitable donors and NFSMAs in the BHJ active layer and by device optimization,^28−30^ with values greater than 26% possible for indoor OSCs.^31^ However, although highly performing nonfullerene acceptors (i.e., the ITIC and Y6 families) are currently available, more work is needed to discover new families of NFSMAs with properties that increase the *V*_OC_, *J*_SC_, and FF values of the devices*.*

Porphyrins are planar and highly conjugated macrocycles that play crucial roles in photosynthesis and other biological processes,^32^ exhibiting remarkable light-harvesting ability as they absorb light in both the blue and red regions of the visible spectrum. In addition, their optical and electrochemical properties can be adjusted by molecular design and functionalization on the β or meso positions of the porphyrin ring as well as by introduction of different central metal ions. Thus, porphyrin derivatives are among the most relevant sensitizers for dye-sensitized solar cells,^33,34^ with efficiencies of up to 14%.^35,36^ However, the pioneering use of porphyrins in OSCs was disappointing as reported efficiencies were very low;^37^ the situation has changed over the past 5 years as Zn-porphyrin derivatives have been applied as donors and PC_71_BM or nonfullerene small molecules have been applied as acceptors, resulting in efficiencies of up to 12% in binary OSCs^38,39^ and 15% in ternary OSCs.^40^

Nevertheless, porphyrin-based compounds acting as acceptor units in OSCs are rarely reported, with the connection of strong electron-withdrawing moieties to the electron-donor Zn-porphyrin macrocycle being the strategy adopted.^41^ In 2014, two different porphyrin derivatives with two isoindigo as end-capped acceptor units were applied as nonfullerene acceptors in solution-processed solar cells paired with P3HT, and the OSCs showed a PCE of approximately 0.57%.^42^ Considering the efficient electron transport processes of porphyrins in natural systems, the acceptor behavior of porphyrins can be improved by judicious molecular design. Li et al. designed a star-shaped electron acceptor compound formed by a porphyrin core linked to four units of perylene bis-imide as end groups and applied it as a nonfullerene acceptor along with the polymer donor PBDB-T for PSCs (polymer solar cells) and achieved a PCE of 7.4%.^43^ Recently, Wang et al. synthesized a series of porphyrin-based electron acceptor molecules and combined them with PTB7-Th as the electron donor for the BHJ active layer and achieved a maximum PCE of 4.28%.^44^ Hadmojo et al. synthesized an electron acceptor molecule based on a Zn-porphyrin linked to four naphthalenediimide units using an ethyne bridge. When paired with a medium-bandgap polymer donor (PTB7-Th), the OSCs showed a PCE of 8.15% and an energy loss of 0.61 eV.^45^ More recently, Belisle et al. described an all-porphyrin OSC with a PCE of 7.2%.^46^ Finally, a series of n-type Zn-porphyrins coupled with 2FIC as strong electron-withdrawing units and different functional groups at the meso positions were reported, affording 7.23% as the best PCE in devices using PTB7-Th as the donor.^47^ Zhu et al. reported the highest PCE reached to date (9.64%) in devices using PTB7-Th as the donor and Zn-porphyrin incorporating four PDI units as the acceptor.^48^ It should be remarked that in all these molecules, the Zn-Por is the electron-donor moiety.

Although the highest occupied molecular orbital (HOMO) of metalloporphyrins can be modified by changing the central metal, most Zn-porphyrins have been studied in OSCs, and other metalloporphyrins have scarcely been used in this field, reaching reasonable efficiencies. These studies were limited to preparing new donors with the aim of obtaining higher *V*_OC_ values by decreasing the HOMO energy of the porphyrin donor.^49,50^

Since the pioneering work by Sauvage et al.,^51^ Au(III) porphyrins have often been used as electron-accepting chromophores in donor–acceptor complexes for the study of photoinduced electron transfer due to their good stability and suitable redox properties.^52,53^ The triplet state of Au(III) porphyrin is directly formed by irradiation with a yield of unity^52,54^ and a lifetime of approximately 50 ns at room temperature.^55^

**Chart 1:**
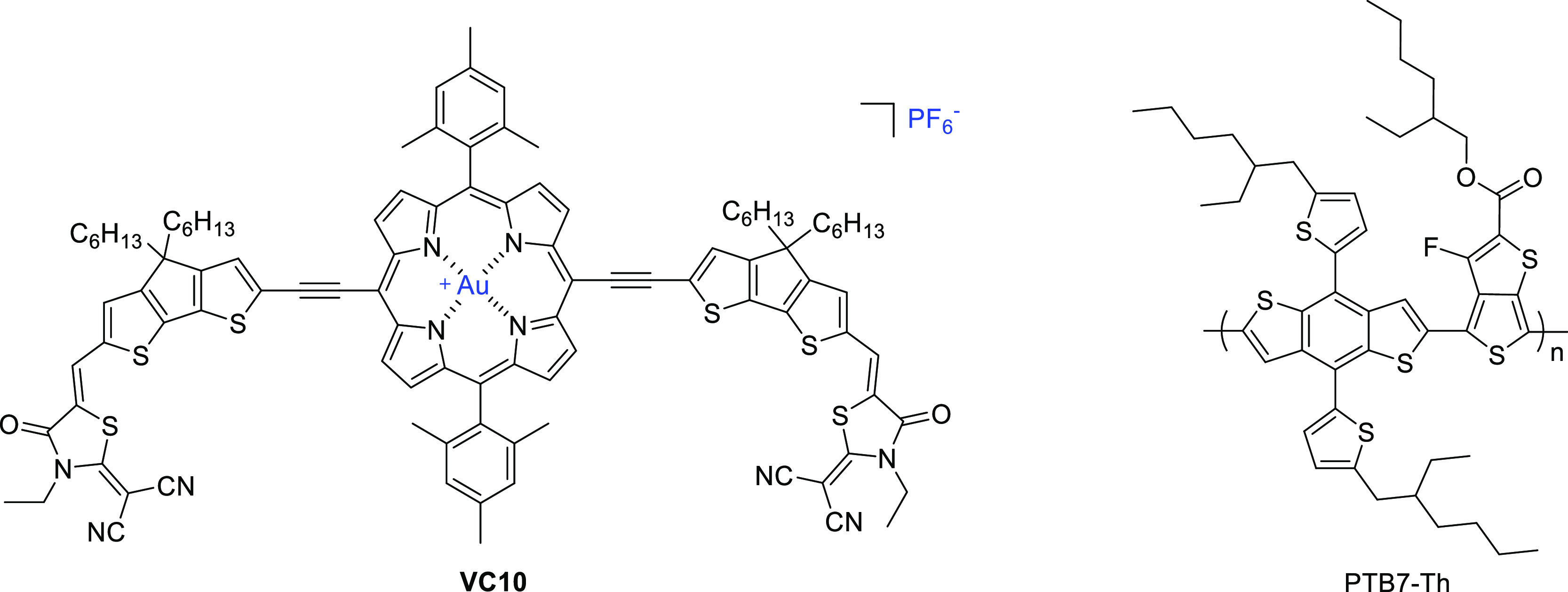
Chemical Structures of **VC10** and PTB7-Th

These results inspired us to examine the effects of a heavy metal atom on the photovoltaic properties of Au(III) porphyrins acting as an acceptor unit in BHJ OSCs. In this work, we designed a new gold porphyrin-based acceptor (**VC10**) with an A_2_-D-A_1_-D-A_2_ skeleton, where A_1_ is a Au(III) porphyrin, A_2_ are the terminal groups of dicyanorhodanine moieties, and the linkers (D) are cyclopentadithiophene (CPDT) groups that exhibit broad absorption spectra covering the visible-to-NIR region of the solar spectrum. A narrow optical bandgap of 1.39 eV and good electron transport properties were achieved due to the strong intramolecular charge transfer (ICT) between Au-Por and acceptor terminals. Using **VC10** as an electron acceptor and PTB7-Th^56^ as the donor (structures are displayed in Chart 1), we achieved an overall PCE of 9.24% and a low energy loss of 0.56 eV after simple optimization of the blend morphology by solvent vapor annealing (SVA).

## Results and Discussion

### Synthesis

**Scheme 1:**
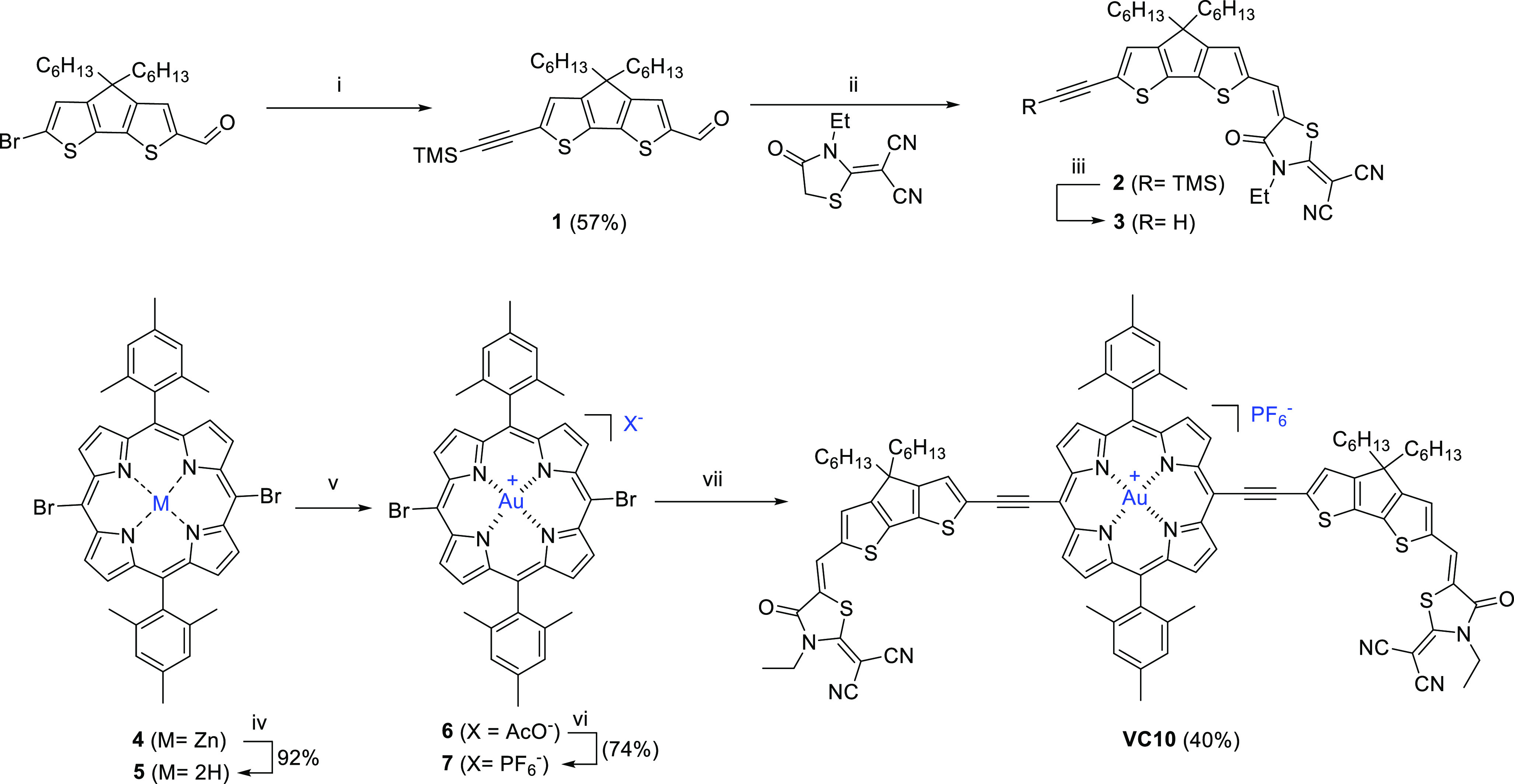
Synthetic Pathway of **VC10** (i) Pd_2_(dba)_3_, AsPh_3_, trimethylsilyl acetylene, Et_3_N/THF (60 °C); (ii) CH_3_COONH_4_/CH_3_COOH (105 °C); (iii) K_2_CO_3,_ MeOH/CHCl_3_; (iv) TFA, CH_2_Cl_2_; (v) KAuCl_4_/AgOTf/CH_3_CO_2_Na, CH_2_Cl_2_/THF (70 °C); (vi) KPF_6_, CH_2_Cl_2_/H_2_O; (vii) **3**, Pd_2_(dba)_3_/AsPh_3_, CH_2_Cl_2_/MeOH, Et_3_N (50 °C).

The synthetic procedure for the Au(III) porphyrin-based molecule **VC10** is shown in Scheme 1. The key precursor Zn-porphyrin **4** was prepared according to the literature procedure.^57^ Transformation of **4** in **7** requires demetallation, followed by reaction with KAuCl_4_/AgOTf/CH_3_CO_2_Na to yield **6**;^58^ the inert contra-ion PF_6_^–^ was incorporated by reaction with KPF_6_ to yield the scaffold Au(III) porphyrin **7**.

Compound **2** was prepared (Scheme 1) from 6-bromo-4,4-dihexyl-4*H*-cyclopenta[2,1-b:3,4-*b*′]dithiophene-2-carbaldehyde^59^ by a Sonogashira reaction with trimethylsilylacetylene, catalyzed by Pd_2_(dba)_3_ and AsPh_3_, followed by Knoevenagel condensation with dicyanorhodanine.^60^

The target compound **VC10** was synthesized in 40% yield by in situ deprotection of the triple bond of **2** using K_2_CO_3_ to afford **3**, followed by the Cu-free Sonogashira coupling of **3** with **7** catalyzed by Pd_2_(dba)_3_·CHCl_3_/AsPh_3_. All new compounds, including **VC10**, were characterized by NMR and MALDI-TOF mass spectrometry (see the experimental details and spectra in the Supporting Information). The molecule shows good thermal stability, with a decomposition temperature of 424 °C (Figure S13: TGA plot of **VC10**), thus proving its aptness for fabrication in photovoltaic devices.^61^

### Optical Properties

**Figure 1:**
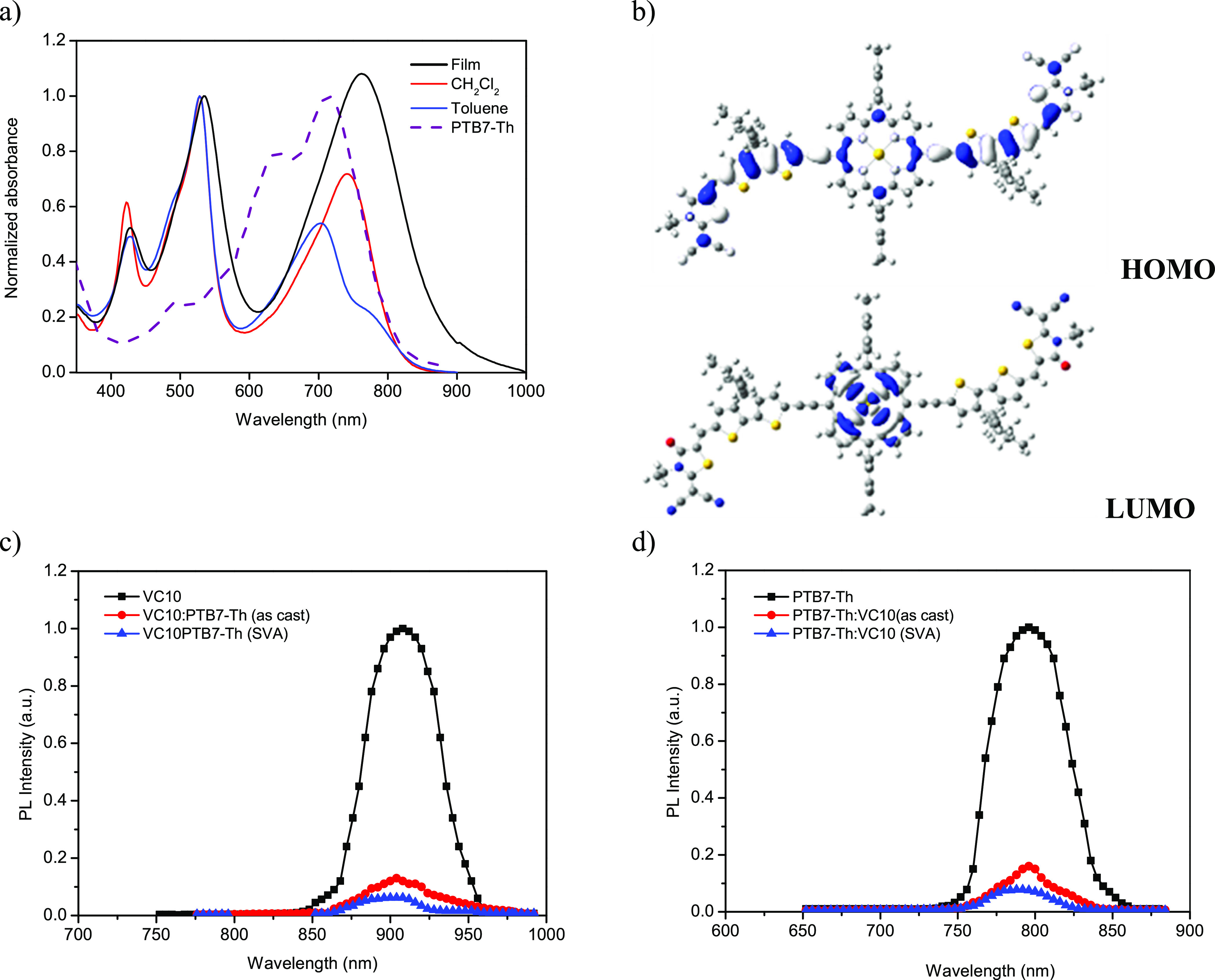
(a) Absorption spectra of **VC10** in solutions (normalized to the Soret porphyrin band) of toluene (blue trace) and CH_2_Cl_2_ (red trace) and in the film (black trace) and PTB7-Th in the film (purple trace). (b) HOMO and LUMO of **VC10**. (c) PL spectra of pristine **VC10** and **VC10**:PTB7-Th (as-cast and SVA-treated). (d) PL spectra of pristine PTB7-Th and PTB7-Th:**VC10** (as-cast and SVA-treated).

The absorption spectra of **VC10** solutions in toluene, dichloromethane, and solid films are shown in Figure 1a, and the data are summarized in Table 1.

**Table 1:** Optical and Redox Properties of **VC10**

	λ_max_/nm (log ε) (sol)^a^	λ_max_/nm (film)	*E*^1^_ox_ (V)^b^	*E*^1^_red_ (V)^b^	*E*_HOMO_ (eV)^c^	*E*_LUMO_ (eV)^d^	*E*_g_ (eV)^c^
**VC10**	422 (4.9)	428	+0.69	–0.89	–5.80	–4.21	1.60
	528 (5.1)	536					
	742 (5.0)	762					

a 10^–6^ M in dichloromethane.

b Conditions: 3.5 × 10^–4^ M in *o*-DCB:acetonitrile (4:1) versus Fc/Fc^+^ (*E*_ox_ = 0.05 V) glassy carbon, Pt counter electrode, 20 °C, 0.1 M Bu_4_NClO_4_, scan rate = 100 mV s^–1^.

c Estimated from *E*_HOMO_ = −5.1 – *E*^1^_ox_; estimated from *E*_LUMO_ = −5.1 – *E*^1^_red_.

d *E*_g_ = *E*_HOMO_ – *E*_LUMO_.

The absorption spectrum of the CH_2_Cl_2_ solution reveals broad and strong Soret and Q bands with maximum peaks at 528 nm (log ε = 5.1) and a wide band ranging from 600 nm to almost 1000 nm, with a peak at 742 nm with a high ε (log ε = 5.0).

The hypsochromic and hypochromic effects observed for the Q band (see Figure 1a) when less polar toluene was used as a solvent indicate the ICT character of this band. Compared to that of diluted solutions, the Q band of the film exhibits a striking redshift (20 nm with respect to that in the dichloromethane solution) and enhanced intensity that can be attributed to *J*-aggregation in the film; the optical bandgap is 1.39 eV. The donor polymer PTB7-Th gave rise to an absorption in the λ = 500–800 nm range, perfectly complementary to that of **VC10**.

The frontier molecular orbital (FMO) energy levels of **VC10** were investigated through electrochemical Osteryoung square wave voltammetry (OSWV) (Table 1 and Figures S14 and S15: anodic and cathodic windows of **VC10**), which identified the HOMO and lowest unoccupied molecular orbital (LUMO) levels at −5.80 and −4.21 eV, respectively. The deep HOMO energy level was also beneficial for air stability. As a comparison, the donor polymer PTB7-Th showed HOMO and LUMO levels of −5.28 and −3.50 eV,^62^ with HOMO and LUMO offsets between **VC10** and PTB7-Th suitable for electron transfer from the LUMO level of PTB7-Th to the LUMO of **VC10** and hole transfer to/from the HOMO energy level of **VC10** to the HOMO energy level of PTB7-Th after the generation of excitons in the active layer.

We used density function theory (DFT) calculations to analyze the geometry and FMO energies of **VC10** and found an almost planar geometry along the conjugated system (Figure 1b and Figure S16: optimized geometry of **VC10**). Additionally, the HOMO was mainly situated at the CPDT moieties with some participation of the porphyrin macrocycle, and the LUMO was localized at the porphyrin core (Figure 1b).

We measured the photoluminescence (PL) spectra of pristine PTB7-Th, **VC10**, and PTB7-Th:**VC10**. Both pristine PTB7-Th and **VC10** showed strong PL emission peaks at 796 and 908 nm when excited at 710 and 770 nm, respectively (Figure 1c,d). The PL intensities of both PTB7-Th and **VC10** were quenched significantly for the PTB7-Th:**VC10** blend and further quenched after SVA treatment. The PL quenching by photoexcitation at 710 nm was attributed to electron transfer from PTB7-Th to **VC10**, whereas the PL quenching by photoexcitation at 776 nm was attributed to hole transfer from **VC10** to PTB7-Th. PL quenching is more noteworthy for the SVA-treated blend at both excitation wavelengths, indicating that charge transfer in the BHJ blend is more efficient than that in the as-cast blend, possibly because of a more favorable nanoscale morphology.

### Photovoltaic Properties

The photovoltaic performance was investigated using the BHJ active layer consisting of PTB7-Th and **VC10** by employing a conventional device structure, i.e., ITO/PEDOT:PSS/active layer/PFN/Al. We selected PTB7-Th as the polymer donor since it exhibits absorption complementary to that of **VC10**, indicating panchromatic absorption by the PTB7-Th:**VC10** active layer, which is beneficial for the light-harvesting efficiency of the OSCs. Initially, we investigated the photovoltaic performance of OSCs by varying the weight ratios between PTB7-Th and **VC10** using benzonitrile as the processing solvent and found that PTB7-Th:**VC10** (1:1.2) showed the best photovoltaic performance.

**Figure 2:**
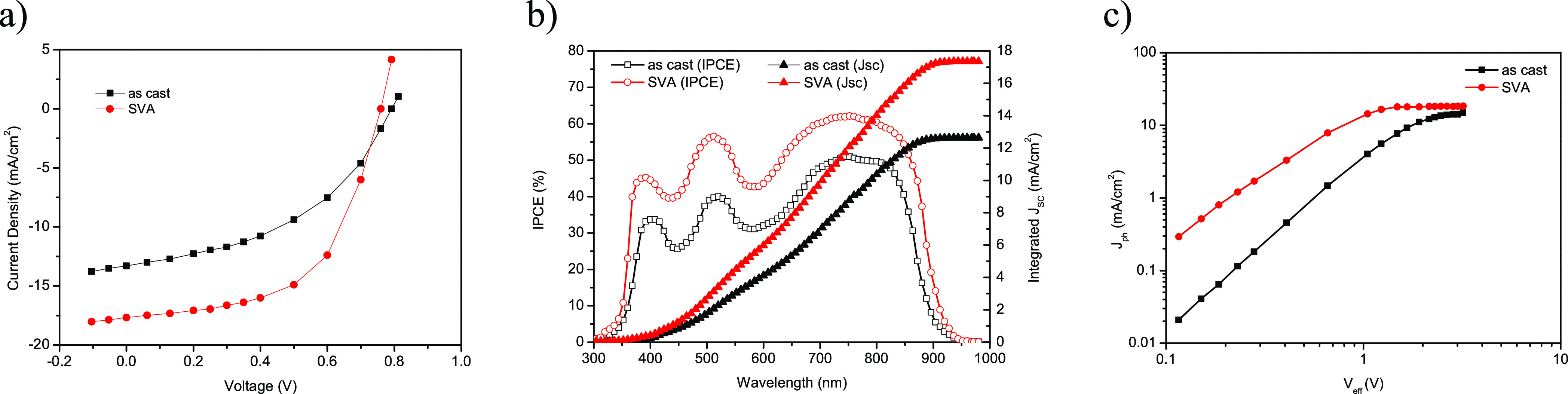
(a) *J*–*V* plots and (b) IPCE of the PTB7-Th:**VC10** active layer as cast and prepared using the SVA technique. (c) Variation in the photocurrent density (*J*_ph_) with effective voltage (*V*_eff_) for OSCs based on as-cast and SVA-treated active layers.

After that, the PTB7-Th:**VC10** (1:1.2) active layer was subjected to SVA for different exposure times in a THF environment, and the OSCs based on the active layer exposed for 40 s showed the best photovoltaic performance. The detailed photovoltaic results of the OSCs are presented in Table S1 (see the Supporting Information). Figure 2a shows the current density–voltage (*J*–*V*) characteristics of the as-cast and SVA-treated OSCs under AM1.5G illumination, and the photovoltaic parameters are summarized in Table 2. The as-cast OSC showed an overall PCE of 6.13% (*J*_SC_ = 13.30 mA/cm^2^, *V*_OC_ = 0.87 V, and FF = 0.53), which increased after SVA treatment to 9.24% (*J*_SC_ = 17.67 mA/cm^2^, *V*_OC_ = 0.83 V, and FF = 0.63), demonstrating the effectiveness of the SVA treatment in this blend.

**Table 2:** Characteristics of the Optimized OSCs of PTB7-Th:**VC10** (1:1.2) Fabricated from Benzonitrile

	*J*_SC_ (mA/cm^2^)	*J*_SC_ (mA/cm^2^)^b^	*V*_OC_ (V)	FF	PCE (%)
as-cast	13.30	13.08	0.87	0.53	6.13 (6.02)^a^
SVA^c^	17.67	17.36	0.83	0.63	9.24 (9.08)^a^

a In brackets, the average of eight devices.

b Estimated from the integration of IPCE.

c SVA in THF for 40 s.

The incident photon-to-current conversion efficiency (IPCE) spectra of the OSCs based on as-cast and SVA-treated active layers are shown in Figure 2b. The OSC based on PTB7-Th:**VC10** shows a broadband photoresponse from 300 to 900 nm, which closely resembles the absorption spectra of the active layer of PTB7-Th:**VC10** (Figure 1a), indicating that the absorption of photons by both PTB7-Th and **VC10** contributes to photocurrent generation. The OSC based on the as-cast active layer showed a maximum IPCE peak of approximately 50%, an increase of up to 62% after SVA treatment of the active layer. The *J*_SC_ values of the OSC estimated from the integration of IPCE spectra were 13.08 mA/cm^2^ (as-cast) and 17.36 mA/cm^2^ (SVA-treated), which are in good agreement with the value obtained from *J*–*V* characteristics under illumination.

By comparing the photovoltaic performance of the as-cast and SVA-treated OSCs, it was found that the SVA treatment of the active layer significantly increased both *J*_SC_ and FF, thereby improving the PCE. To understand the increase in *J*_SC_ and FF after SVA treatment, we examined the relationship between the photocurrent density (*J*_ph_) and effective voltage (*V*_eff_) of the OSCs and investigated the exciton dissociation probability (*P*_diss_) and charge collection probability (*P*_coll_) in these two PSCs.^63^Figure 2c shows that with the initial increase in *J*_ph_ with increasing *V*_eff_, *J*_ph_ reaches saturation (*J*_sat_) at a high value of *V*_eff_. The *J*_ph_ value for the as-cast OSC reaches saturation at a much higher *V*_eff_ (2.4 V) than that for the SVA-treated counterpart (1.2 V).

The values of *P*_diss_ and *P*_coll_ were estimated as *J*_ph_/*J*_sat_ under short circuit conditions and maximum power points, respectively. The values of *P*_diss_/*P*_coll_ were 0.89/0.68 and 0.96/0.75 for the as-cast and SVA-treated OSCs, respectively, indicating that the SVA treatment of the active layer increased both the exciton dissociation and the charge collection. The maximum exciton generation rate (*G*_max_) was estimated as *G*_max_ = *J*_sat_/*qL*, where *q* is the electronic charge and *L* is the thickness of the active layer. The values of *G*_max_ were approximately 0.98 × 10^28^ and 1.21 × 10^28^ m^–3^ s^–1^. The higher value of *G*_max_ for OSC based on the SVA treatment is consistent with the larger values of IPCEs and *J*_SC_.

We examined the hole mobility and electron mobility of the PTB7-Th:**VC10** active layer by measuring the dark *J*–*V* characteristics of hole- and electron-only devices, respectively, and fitting them with a space charge limited current model (Figure S17a,b: dark *J*–*V* characteristics of hole-only and electron-only devices using PTB7-Th:**VC10** films).^64^ The hole and electron mobilities for the as-cast device were 3.67 × 10^–4^ and 1.24 × 10^–4^ cm^2^/V s, respectively, with a hole-to-electron mobility ratio of 2.96. After the SVA treatment, the hole and electron mobilities increased to 5.13 × 10^–4^ and 4.23 × 10^–4^ cm^2^/V s, respectively, with a hole-to-electron mobility ratio of 1.24, indicating more balanced charge transfer, which is beneficial for the improvement in *J*_SC_ and FF.

To obtain more information about the recombination processes in the active layer, we examined the variation in *J*_SC_ and *V*_OC_ with the illumination intensity (*P*_in_) of the OSCs,^65,66^ and this is shown in Figure S18a,b (variation in *J*_SC_ and *V*_OC_ for the OSCs based on PTB7-Th:**VC10**).

The variation in *J*_SC_ with *P*_in_ followed the relationship *J*_SC_ ∝ (*P*_in_)^α^, where α is related to the degree of bimolecular recombination and must be equal to unity for an imperceptible bimolecular recombination. The values of α for the as-cast and SVA-based devices were 0.91 and 0.954, respectively, indicating that SVA treatment effectively suppressed bimolecular recombination. The variation in *V*_OC_ values with *P*_in_ was recorded to examine trap-assisted recombination in the active layer of the PSCs, and it is shown in Figure 2b. The relationship between *V*_OC_ and *P*_in_ can be expressed as *V*_OC_ = (*nkT*/*q*) ln(*P*_in_). The value of *n* describes the degree of trap-assisted recombination. The values of *n* for as-cast and SVA-treated PTB7-Th:**VC10** were approximately 1.39 and 1.28, respectively, indicating that SVA treatment of the active layer suppressed trap-assisted recombination due to the more favorable nanoscale phase separation in the active layer after SVA treatment.

Moreover, the energy loss (*E*_loss_) was defined as *E*_loss_ = *E*_g_ – *qV*_oc_, where *E*_g_ is the onset of the IPCE spectra of the OSC.^67^ The estimated *E*_loss_ for PSCs based on the optimized PTB7-Th:**VC10** was approximately 0.52 eV, which is the smallest *E*_loss_ described for PSCs based on porphyrin acceptors.

### Active Layer Morphology

**Figure 3:**
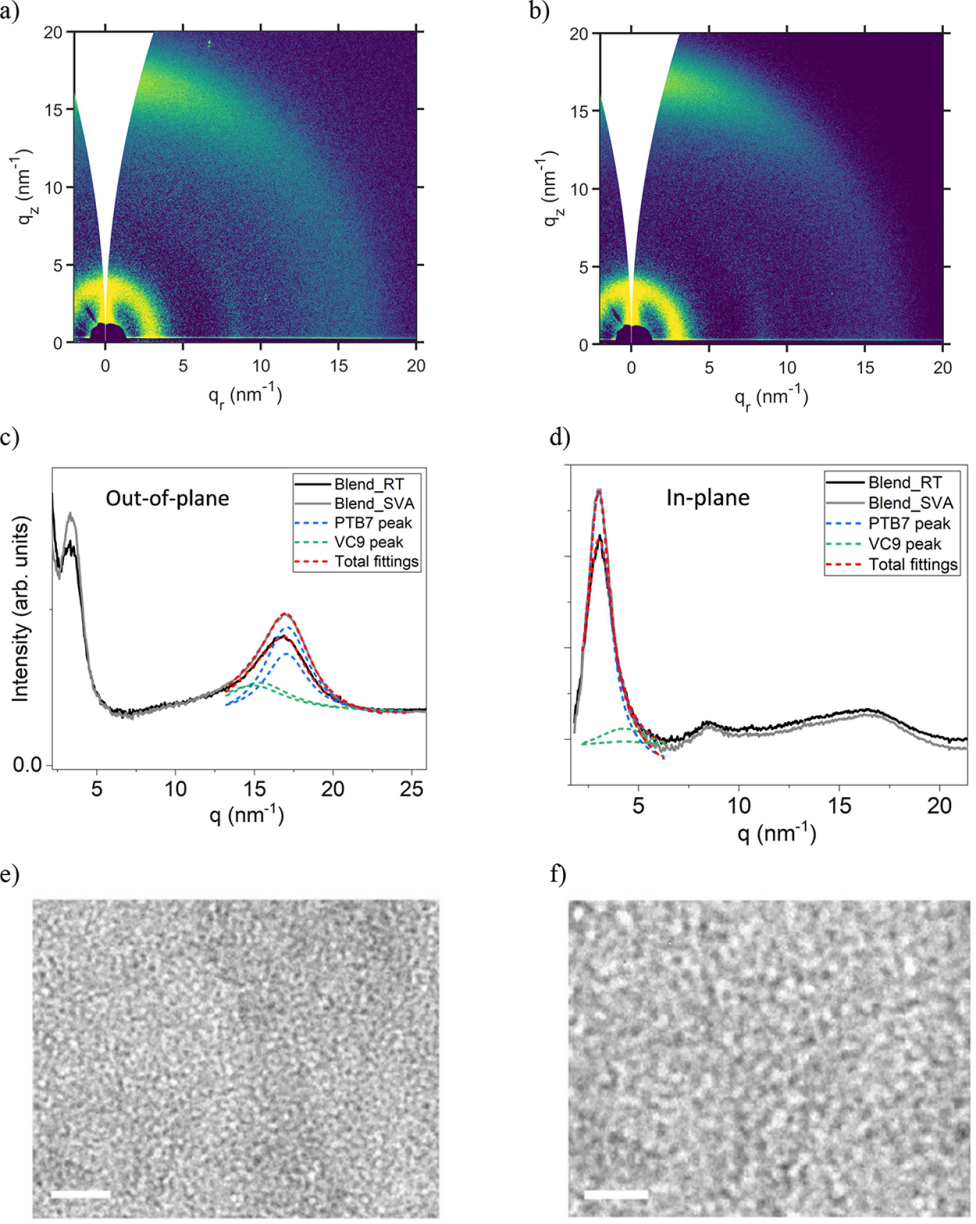
(a, b) GIWAXS patterns of the as-cast and SVA-treated blend samples. (c, d) Gray and black curves correspond to the intensity profiles obtained by azimuthal integrations of the GIWAXS patterns along the *q*_z_ axis (out-of-plane) and along the *q*_r_ axis (in-plane). Red curves are total scattering intensity resulting from fitting experimental signals to contributions from PTB7-Th (blue lines) and **VC10** (green lines). TEM images of (e) as-cast and (f) SVA-treated PTB7-Th:**VC10** thin films (scale bars: 100 nm).

The structural order of the BHJs was investigated by grazing incident wide angle X-ray scattering (GIWAXS).^68−70^ The GIWAXS patterns shown in Figure 3 reveal a few differences between the as-cast and SVA-treated blends from a microstructural viewpoint. Because of the amorphous structure of **VC10** (see the Supporting Information, Figure S20), the patterns for the blends displayed mainly the peaks from PTB7 molecular aggregates, i.e., the (100) and (010) peaks associated with the lamellar order and π–π stacked planes, respectively. Both peaks were slightly narrower for the SVA-treated sample (peak analysis is included in the Supporting Information, Figure S19: GIWAXS patterns from the PTB7 samples; Figure S20: GIWAXS patterns from the **VC10** samples), indicating a slightly enhanced order in the latter, but the difference was too minor to explain the improvement in the photovoltaic performance. Indeed, further results included in the Supporting Information (Figures S19 and S20) suggest that SVA treatment generally has little impact even on the structure of the individual, nonmixed materials.

SVA treatment, however, had a major impact on the blend nanomorphology. Shown in Figure 3 are representative transmission electron microscopy (TEM) images of the as-cast (Figure 3e) and SVA-treated (Figure 3f) PTB7-Th:**VC10** blends. Due to their lower and higher electron densities, the bright and dark zones correspond to the donor- and acceptor-rich domains, respectively. As the domain size of the donor and acceptor in the as-cast film is small, it may hinder the charge transport for electrons to holes toward the cathode (Al electrode) and anode (ITO) and result in a low value of FF. Clearly, the domains become larger after SVA treatment, resulting in a reduced bimolecular recombination rate and thus agreeing with the observed increase in FF. As the series resistance values of the as-cast and SVA-treated devices are 4.58 and 2.51 Ω cm, respectively (estimated from the *J*–*V* curves; Figure 2a), which also support the increased value of FF for SVA-treated OSC. The improved charge transfer of blends after SVA treatment suggests that the resulting domains exhibit a higher degree of percolation. Altogether, these morphological changes allow rationalization of the enhancement of the PCE observed after SVA treatment.

## Conclusions

In conclusion, in recent years, many Zn-porphyrin-based donors have been developed to produce highly efficient OSCs. Nevertheless, a few examples of porphyrin-based acceptors, mainly Zn-porphyrins containing perylene diimide groups, can be found in the literature and usually display low efficiencies, demonstrating that more work is necessary in this important field. In this work, we designed and synthesized Au(III) porphyrin-based materials with two CPDT-dicyanorhodanine arms for the first time, showing broad absorption up to 920 nm and a high absorption coefficient and optical bandgap of 1.39 eV. This molecule has been used as a nonfullerene acceptor in OSCs blended with a conjugated polymer with complementary absorption as a donor, providing an excellent PCE of 9.24% and an energy loss of 0.52 eV after SVA treatment. This result demonstrates that this structural design can be used to synthesize a new generation of nonfullerene acceptors based on gold porphyrins, providing new guidelines for molecular engineering of the central metal of porphyrin with the aim of constructing porphyrin-based acceptors. We are working now on the synthesis of new Au-porphyrins with more adjusted FMOs to achieve even higher efficiencies in OSCs.

## Experimental Section

### Synthesis of Compound **1**

In a Schlenk flask under an Ar atmosphere, 6-bromo-4,4-dihexyl-4*H*-cyclopenta[2,1-*b*:3,4-*b*′]dithiophene-2-carbaldehyde^59^ (2.2 mmol, 1 g), Pd_2_(dba)_3_ (1.32 mmol, 1.2 g), and AsPh_3_ (8.8 mmol, 2.7 g) were kept under vacuum for 1 h. Then, trimethylsilyl acetylene (8.82 mmol, 1.42 g) was added to the mixture and solved in dry THF (140 mL) after freshly distilled Et_3_N (30 mL) was added. The reaction was stirred overnight at 60 °C. The crude was filtrated by Celite, and the solvent was removed under reduced pressure. A chromatographic column was carried out on silica gel with a mixture of hexane:CHCl_3_ (from 9:1 to 7:3). Compound **1** was obtained as a dark oil (600 mg, 1.27 mmol, yield: 57%). ^1^H-NMR (400 MHz, CDCl_3_) δ/ppm: 9.77 (s, 1H), 7.50 (s, 1H), 7.19 (s, 1H), 1.79–1.74 (m, 4H), 1.10–1.03 (m, 8H), 0.82–0.73 (m, 14H), 0.20 (s, 9H).

### Synthesis of Compound **2**

In a round-bottom flask, to a mixture of **1** (0.64 mmol, 305 mg), 2-(1,1-dicyanomethylene)-3-ethyl-rhodanine^60^ (1.61 mmol, 312 mg), and AcNH_4_ (1.7 mmol, 131 mg), glacial acetic acid (9 mL) was added. The mixture was stirred at 105 °C for 4 h. The product was purified by preparative TLC with a mixture of hexane:CH_2_Cl_2_ (from 3:1 to 3:2) as eluent to obtain **2** as a red solid (144 mg; 0.22 mmol; yield: 35%). ^1^H-NMR (400 MHz, CDCl_3_) δ/ppm: 8.09 (s, 1H), 7.42 (s, 1H), 7.14 (s, 1H), 4.34 (d, *J* = 7.2 Hz, 2H), 1.84 (t, *J* = 8.2 Hz, 4H), 1.46–1.41 (m, 3H), 1.19–0.8 (m, 22H), 0.29 (d, *J* = 2.0 Hz, 9H); ^13^C-NMR (100 MHz, CDCl_3_) δ/ppm: 166.1, 165.5, 161.0, 160.9, 146.9, 138.1, 137.1, 130.0, 128.7, 127.5, 127.3, 113.7, 112.7, 111.2, 102.6, 98.2, 54.5, 40.8, 37.9, 31.7, 29.7, 24.7, 22.8, 14.4, 14.2; MALDI-TOF MS (*m*/*z*): calculated for C_35_H_43_ON_3_Si, 645.23 [M^+^]; found, 645.215.

### Synthesis of Compound **3**

To a mixture of compound **2** (104 mg, 0.16 mmol) in MeOH (3 mL) and CHCl_3_ (3 mL), K_2_CO_3_ (111 mg, 0.8 mmol) was added. The reaction was checked by TLC, and when the starting product disappeared (20 min), the reaction was quenched with water. The crude was extracted with CHCl_3_, and the organic phase was dried over Na_2_SO_4_. Compound **3** was used in the next step without further purification.

### Synthesis of Compound **5**

Under an Ar atmosphere to a mixture of **4**(71) (0.58 mmol, 452 mg) in CH_2_Cl_2_ (580 mL), trifluoroacetic acid (TFA) (77.7 mmol, 5.58 mL) was added. The mixture was stirred for 40 min at room temperature. The reaction was quenched with a saturated solution of K_2_CO_3_ (200 mL) and stirred for 20 min. Then, the crude was extracted with CH_2_Cl_2_ (200 mL × 3). The organic phase was dried over Na_2_SO_4_, the solvent was removed under reduced pressure, and the obtained solid was filtered by silica gel in column chromatography with CHCl_3_:hexane (7:3) as eluent. The solid was washed five times by centrifugation with MeOH and pentane to obtain porphyrin **5** as a purple solid (0.53 mmol, 378 mg, 92%). ^1^H-NMR (400 MHz, CDCl_3_) δ/ppm: 9.56 (d, *J* = 4.8 Hz, 4H), 8.71 (d, *J* = 4.8 Hz, 4H), 7.30 (s, 4H), 2.65 (s, 6H), 1.83 (s, 12H), −2.55 (s, 2H). MALDI-TOF MS (*m*/*z*): calculated for C_38_H_32_Br_2_N_4_, 702.10 [M^+^]; found, 702.56.

### Synthesis of Compounds **6** and **7**

In a round-bottom flask, a mixture of **5** (0.36 mmol, 259 mg) and sodium acetate (AcNa) (3.6 mmol, 295 mg) in CH_2_Cl_2_ (20 mL) was stirred at 35 °C for 10 min. Then, a mixture of silver trifluoromethanesulfonate (AgOTf) (3.6 mmol, 924 mg) and KAuCl_4_ (1.84 mmol, 695 mg) in THF (38 mL) was added to the solution. The reaction was stirred overnight at 70 °C. The solvent was removed under reduced pressure, and the obtained solid was dissolved in CHCl_3_ and filtrated on silica gel in 100% CHCl_3_. Without further purification, molecule **6** was dissolved in CH_2_Cl_2_ (32 mL) and mixed with a solution of KPF_6_ (34.5 mmol, 6361 mg) in H_2_O (32 mL). This reaction mixture was stirred overnight at room temperature and afterward extracted with CH_2_Cl_2_ (3 × 30 mL), and the organic phase was dried over Na_2_SO_4_. The solvent was removed under reduced pressure, and the obtained solid was washed five times with pentane to obtain molecule **7** as a red solid (0.26 mmol; 240 mg; yield: 74%). ^1^H-NMR (400 MHz, CDCl_3_) δ/ppm: 10.01 (d, *J* = 5.2 Hz, 4H), 9.16 (d, *J* = 5.2 Hz, 4H), 7.36 (s, 4H), 2.67 (s, 6H), 1.80 (s, 12H). ^13^C-NMR (100 MHz, CDCl_3_) δ/ppm: 139.9, 139.3, 137.4, 137.3, 134.4, 134.2, 132.5, 128.6, 123.5, 21.5, 21.4. ESI-MS (*m*/*z*): positive mode: calculated for C_38_H_30_AuBr_2_N_4_ 897.05 [M^+^]; negative mode: found, 897.32. Calculated for PF_6_^–^, 144.96 [M + 1^+^]; found, 143.2.

### Synthesis of **VC10**

In a Schlenk flask, porphyrin **7** (48 mg, 0.05 mmol), **3** (92 mg, 0.16 mmol), Pd_2_(dba)_3_·CHCl_3_ (7 mg, 0.006 mmol), and AsPh_3_ (5 mg, 0015 mmol) were kept under vacuum for 1 h. Next, the mixture was diluted in dry CH_2_Cl_2_ (7 mL) and MeOH (7 mL), and finally, freshly distilled Et_3_N (0.73 mL) was added. The reaction was stirred for 30 min at 50 °C. Then, the solvent was removed under vacuum. The product was isolated by gel permeation chromatography in CH_2_Cl_2_, and then the product was precipitated in pentane to obtain **VC10** as a dark shiny green solid (40 mg; 0.021 mmol; yield: 40%). ^1^H-NMR (400 MHz, THF-*d*_8_) δ/ppm: 9.98 (d, *J* = 5.1 Hz, 4H), 9.04 (d, *J* = 5.1 Hz, 4H), 8.15 (s, 2H), 8.01 (s, 2H), 7.60 (s, 2H), 7.36 (s, 4H), 4.19 (q, *J* = 7.2 Hz, 4H), 2.57 (s, 6H), 2.36 (d, *J* = 16.0 Hz, 8H), 2.03–2.00 (m, 8H), 1.77 (s, 12H), 1.26 (t, *J* = 8 Hz, 10H), 1.18–1.11 (m, 12H), 0.98 (m, 8H), 0.73 (t, *J* = 6.6 Hz, 12H). ^13^C-NMR (100 MHz, THF-*d*_8_) δ/ppm: 163.7, 163.2, 159.7, 159.6, 143.4, 138.6, 138.2, 137.9, 136.9, 136.6, 134.6, 129.8, 127.6, 127.5, 126.8, 126.7, 123.2, 121.8, 111.4, 110.9, 110.3, 103.0, 53.1, 52.6, 38.4, 35.7, 29.8, 27.8, 20.7, 18.8, 18.5, 11.6, 11.4. FT-IR (ATR) ν/cm^–1^: 2926, 2855, 2216, 2169, 1717. MALDI-TOF MS (*m*/*z*): calculated for C_102_H_98_AuN_10_O_2_S_6_: 1885.30 [M^+^]; found, 1886.05. UV–Vis (CH_2_Cl_2_) λ/nm (log ε): 422 (4.9), 528 (6.1), 742 (5.0).

## Supporting Information Available

General remarks; ^1^H-NMR, ^13^C-NMR, FT-IR, and mass spectrometry spectra of synthesized compounds; TGA and cyclic voltammetry plots of **VC10**; theoretical calculation; photovoltaic and GIWAXS complementary data (PDF)
