# Supplementary material for: Gold(III) Porphyrin Was Used as an Electron Acceptor for Efficient Organic Solar Cells
Source: ACS Appl Mater Interfaces. 2022 Feb 23;14(9):11708–17. doi: 10.1021/acsami.1c22813 (PMC8915169; doi:10.1021/acsami.1c22813)
Supplement: Supplementary file 1 — am1c22813_si_001.pdf [file am1c22813_si_001.pdf]

Supporting Information

Gold(III) porphyrin was used as an electron acceptor for efficient organic solar cells

Virginia Cuesta,[†] Manish Kumar Singh,[§] Edgar Gutierrez-Fernandez,[‡] Jaime Martín,[‡,γ,ξ] Rocio Dominguez,[†] Pilar de la Cruz,[†] Ganesh D. Sharma,[§]* and Fernando Langa[†]*.*

[†] Universidad de Castilla-La Mancha, Institute of Nanoscience, Nanotechnology and Molecular Materials (INAMOL), Campus de la Fábrica de Armas, 45071-Toledo, Spain.

[§] Department of Physics, The LNM Institute of Information Technology (Deemed University), Jamdoli, Jaipur (Raj.) 302031, India.

[‡] POLYMAT, University of the Basque Country UPV/EHU Av. de Tolosa 72, 20018, San Sebastián, Spain

[γ] Ikerbasque Basque Foundation for Science, Bilbao 48013, Spain.

[ξ] Universidade da Coruña, Grupo de Polímeros, Centro de Investigacións Tecnolóxicas (CIT), Esteiro, 15471 Ferrol, Spain.

1. **General Remarks** 2
2. **^{1}H-NMR, ^{13}C-NMR, FT-IR and MS spectra.** 4
3. **Thermogravimetric analysis (TGA) of VC10.** 10
4. **Electrochemical studies.** 11
5. **Theoretical Calculations** 12
6. **Photovoltaic Studies** 13
7. **GIWAX** 16

1. General Remarks

All solvents and reagents were purchased from Aldrich Chemicals. The solvents were used without previous purification. Anhydrous solvents were dried by purification system Pure-Sov 400. Chromatographic purifications were performed using silica gel 60 Merck 230-400 mesh ASTM. Gel Permeation Chromatography (GPC) column was performed using Bio- Beads ® S-X1 Beads 200-400 Mesh as stationary phase. Analytical thin-layer chromatography was performed using ALUGRAM ® SIL G/UV254 silica gel 60. Nuclear magnetic resonance ^{1}H-NMR and ^{13}C-NMR were performed using Bruker Innova 400 Hz. ^{1}H-NMR chemical shifts (*δ)* values are denoted in ppm. Residual solvent peaks have been used as the internal standard ($CHCl_3$: δ = 7.27 ppm; THF-*d8*: δ = 3.58 and 2.54 ppm), ^{13}C-NMR chemical shifts are reported relative to the solvent residual peaks ($CDCl_3$, δ = 77.00 ppm; THF: δ = 67.6 and 1.73 ppm). MALDI-TOF spectra were obtained in a Bruker UltrafleXtreme mass spectrometer, using dithranol [1,8- dihydroxy-9(10H)-anthracenone] as matrix. Fourier transform infrared spectrophotometer (FT-IR) Thermo Nicolet AVATAR 370 was used with KBr pellet, in each case the most characteristic bands are indicated for each compound. Absorption spectra were performed on Shimadzu UV 3600 spectrophotometer. Solutions of different concentration were prepared in solvents with spectroscopy grade, with 0.3 absorbance using a 1 cm UV cuvette. The thermal stability was evaluated by TGA on a Mettler Toledo TGA/DSC STARTe System under nitrogen, with a heating rate of 10 ^{o}C/min.

Electrochemical Measurements: Reduction (E_{red}) and oxidation potentials (E_{ox}) were measured by Cyclic (CV) and Osteryoung Square Wave (OSWV) voltammetries with a potentiostat BAS CV50W in a conventional three-electrode cell equipped with a glassy carbon working electrode, a platinum wire counter electrode and a Ag/$AgNO_3$ reference electrode at scan rate of 100 mV/s. The E_{red} and E_{ox} were expressed vs. Fc/Fc+ used as external reference. In each case, the measurements were done in a deaerated solution containing 1 mM of a the sample compound in 0.1 M of $(n\text{-Bu})_4NClO_4$ in *o*-DCB:Acetonitrile (4:1) as an electrolyte solution.

Computational Details: Theoretical calculations were carried out in the supercomputation service of UCLM within the density functional theory (DFT) framework by using the Gaussian 09, applying density functional theory at the B3LYP level. The basis set of LANL2DZ was used in the calculations.

Fabrication of Photovoltaic device and characterization. The solution processed organic solar cells were fabricated on the ITO coated glass substrate with structure ITO/PESOT:PSS/active layer (P:VC10)/PFN/Al. The ITO coated glass substrates were cleaned in detergent, and subsequently ultra-sonicated in deionized water, acetone and isopropyl alcohol and dried *in vacuum* oven to remove all the traces of residues. The photovoltaic performance optimization process was started with identifying the donor to acceptor ratio (weight percentage, varying from 1:0.4 to 1:1.3) and after that solvent vapor annealing was applied to maximize the performance of the OSCs. The conjugated polymer PTB7-Th was used as acceptor (A) and the total concentration of D:A blend mixture was 16 mg/mL in chloroform. The devices were fabricated by depositing PEDOT:PSS as hole transport layer having thickness of 35-40 nm. The active layer was deposited by spin coating (2500 rpm, 60 s) on the top of PEDOT:PSS layer under ambient conditions. For the solvent vapor annealing (SVA), the optimized (as cast 1:1.5 D/A wt ratio) was exposed to the THF vapors for 40s. A thin layer of PFN was spin coated on the top of the active layer from the methanol solution. The aluminium (Al) electrode was deposited onto the top of PFN layer *via* thermal evaporation at the pressure less than 10^{-5} Torr. The current-voltage characteristics of the OSCs were measured under illumination intensity of 100 mW/cm^2 (AM1.5 G) using a solar simulator and a Keithley 2400 source meter unit. The External quantum efficiency (EQE) measurements were performed using Bentham EQE system.

GIWAXS. GIWAXS experiments were carried out at ALBA Synchrotron (Barcelona, Spain) at the NCD-SWEET beamline. The energy of the incoming beam was set at 12.9 keV (λ=0.0957 nm). The patterns were taken with a WAXS LX255-HS detector (Rayonix) placed at 230 mm from the sample, exposing the detector to the scattered photons no longer than 5 seconds. The data analysis, including the azimuthal integrations and the geometrical corrections of the 2D patterns were performed using self-written Python and MATLAB codes.

2. ^{1}H-NMR, ^{13}C-NMR, FT-IR and MS spectra.

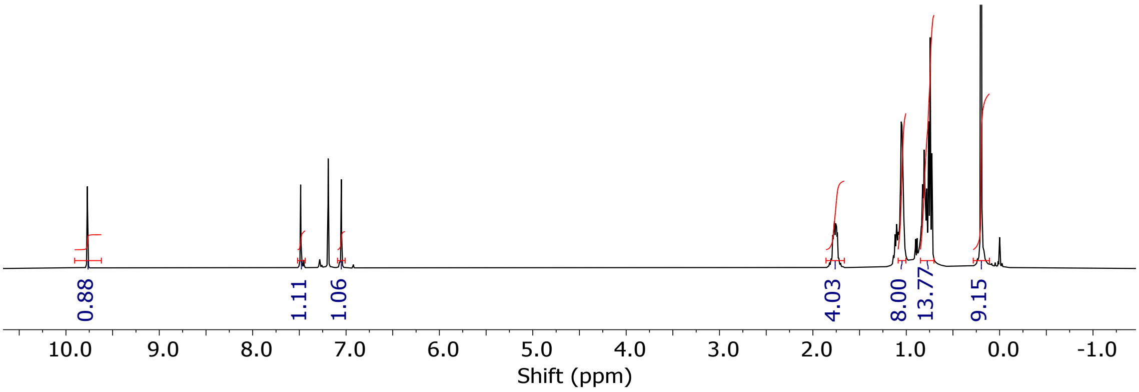

Figure S1. ^{1}H-NMR of compound **1** (400 MHz, $CDCl_3$).

a)

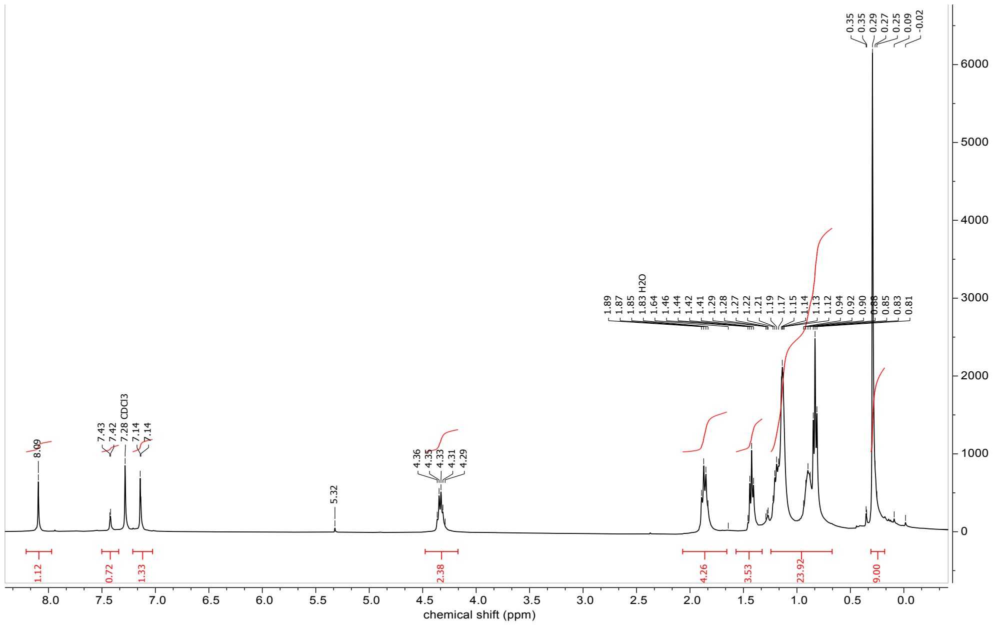

b)

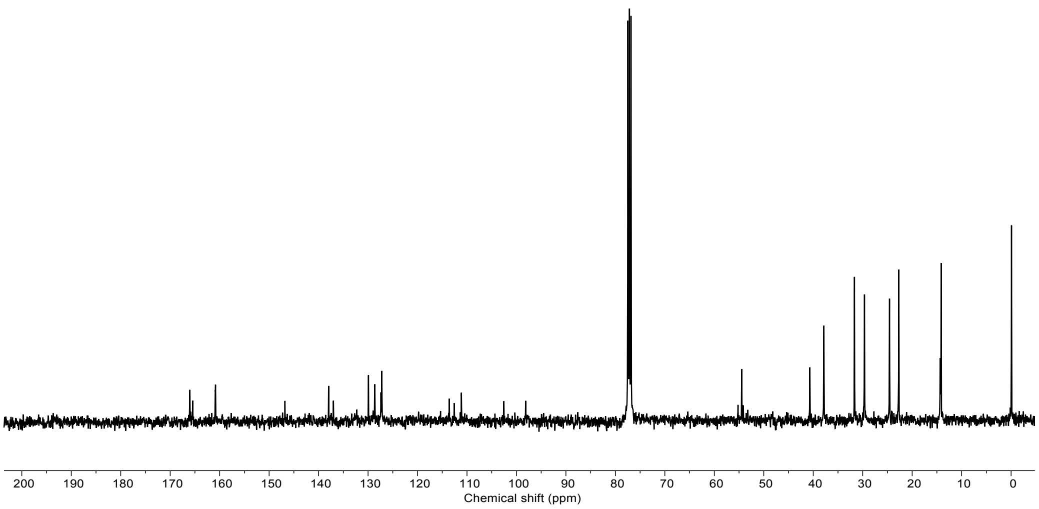

c)

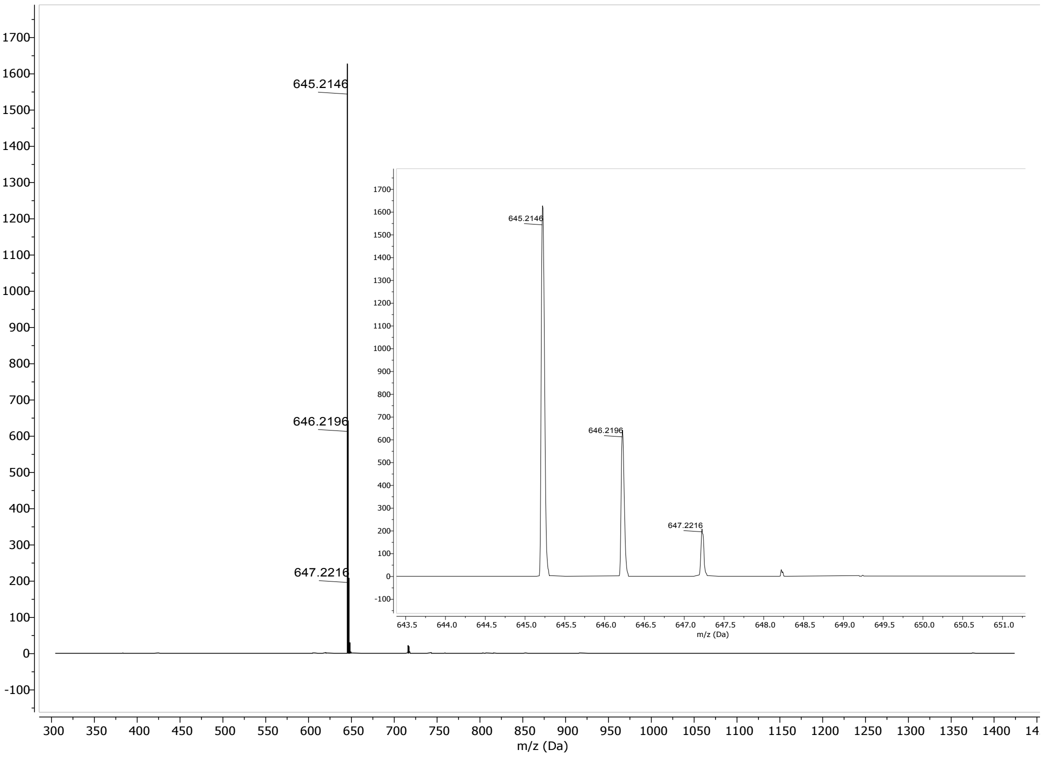

Figure S2. Structural characterization of compound **2**: a)^{1}H-NMR spectrum (400 MHz, $CDCl_3$), b) ^{13}C-NMR spectrum (100 MHz, $CDCl_3$) and c) MALDI-TOF MS spectrum.

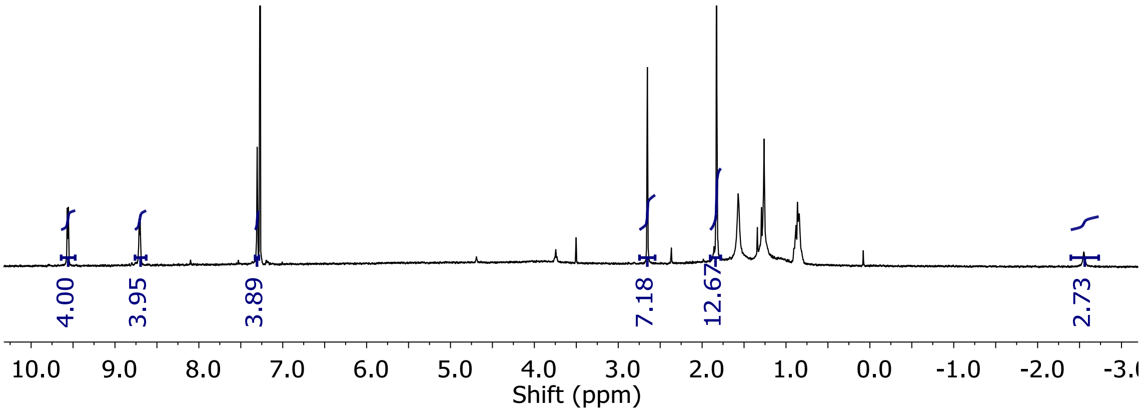

Figure S3. ^{1}H-NMR of compound **5** (400 MHz, $CDCl_3$).

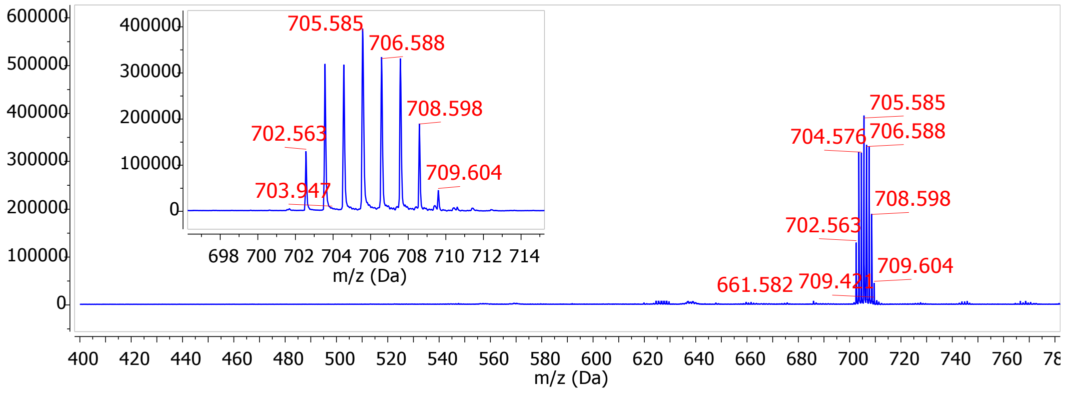

Figure S4. MALDI-TOF MS spectrum of compound **5**.

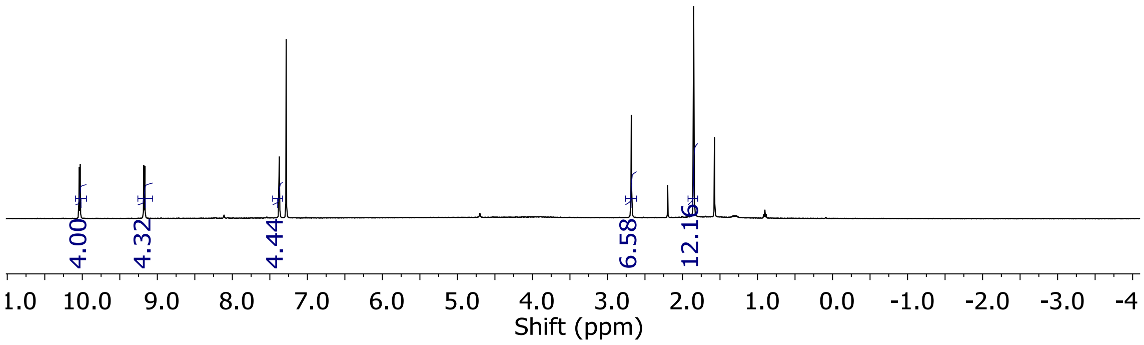

Figure S5. ^{1}H NMR of compound **7** (400 MHz, $CDCl_3$).

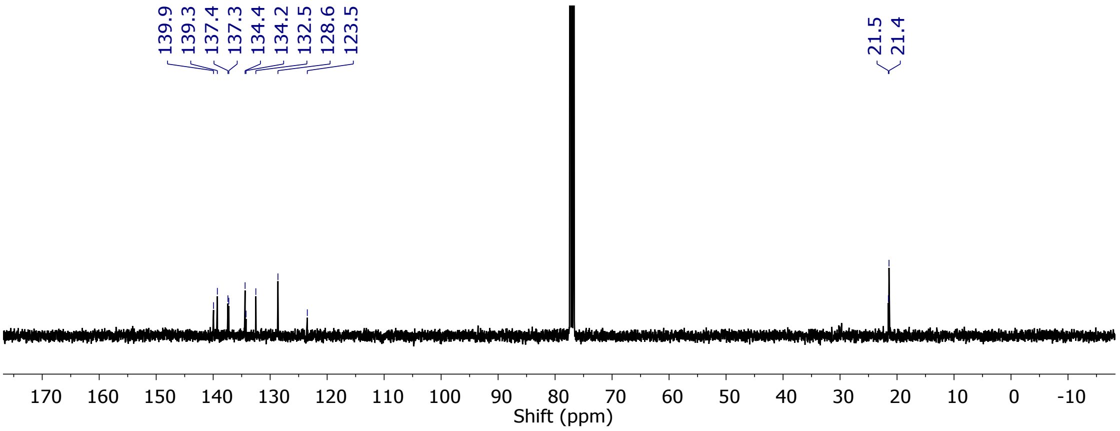

Figure S6. ^{13}C NMR of compound **7** (100 MHz, $CDCl_3$).

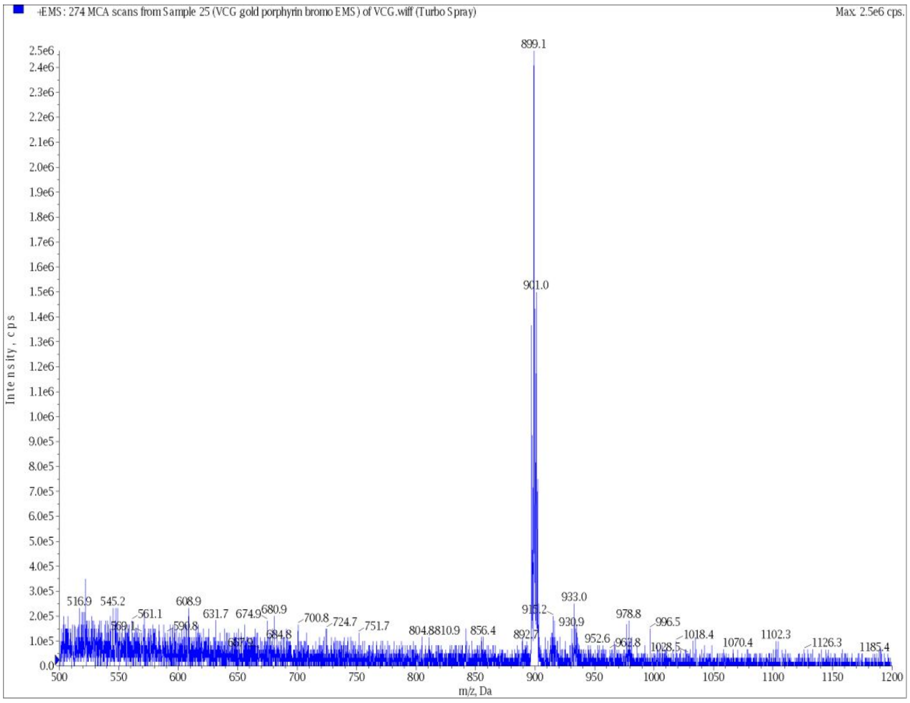

Figure S7. ESI MS spectrum of compound **7** (positive mode).

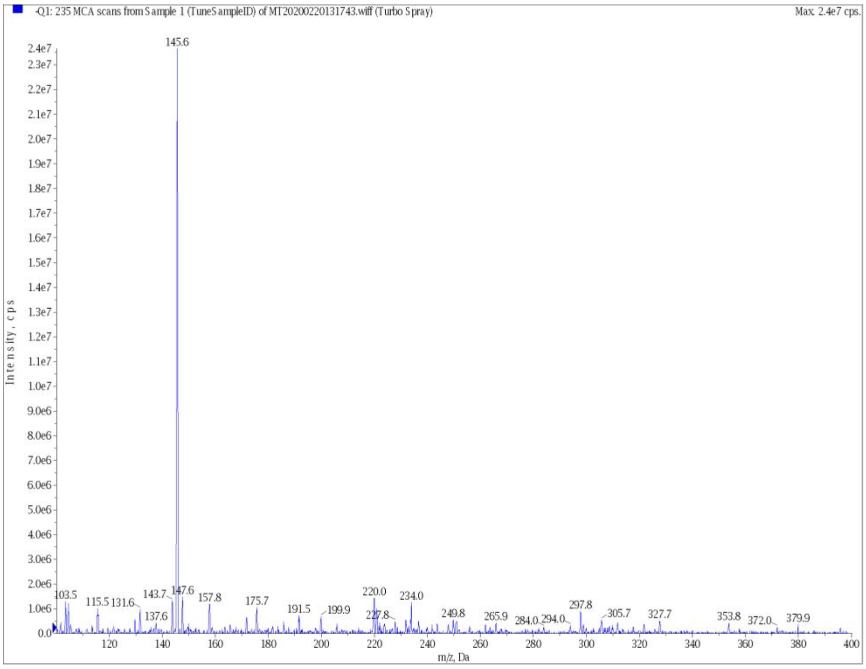

Figure S8. ESI MS spectrum of compound **7** (negative mode).

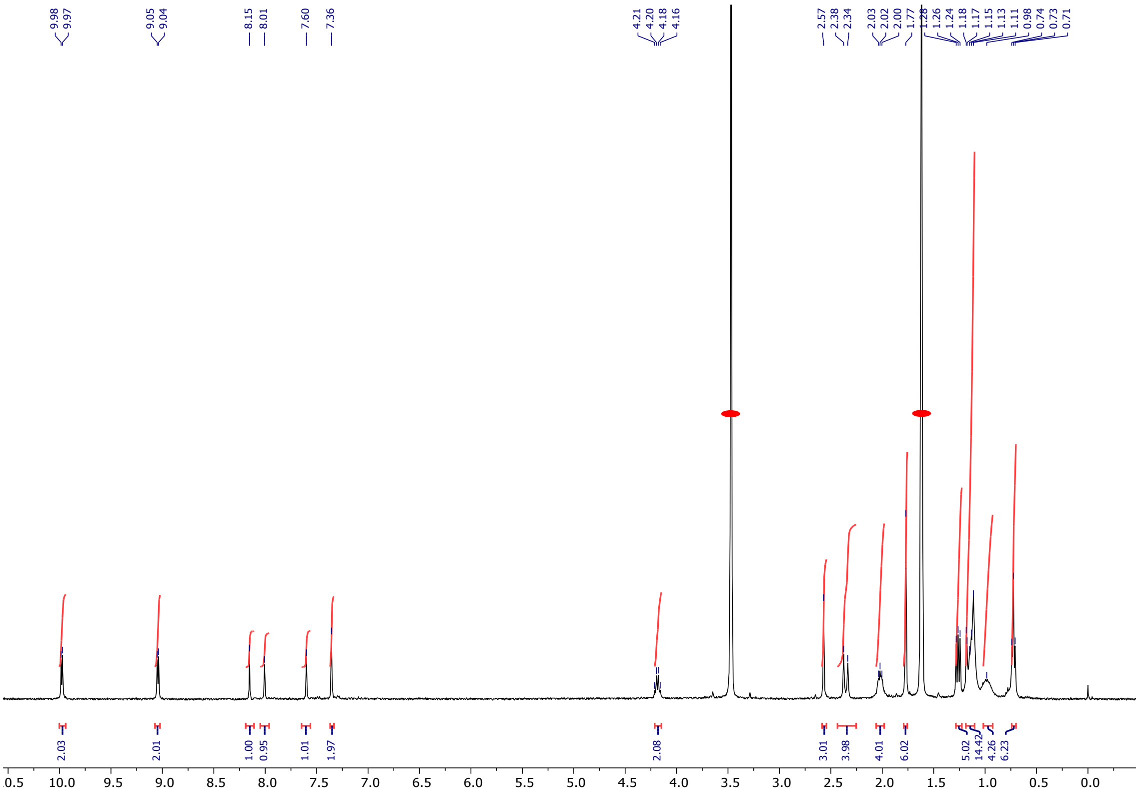

Figure S9. ^{1}H NMR of compound **VC10** (400 MHz, THF-d_8).

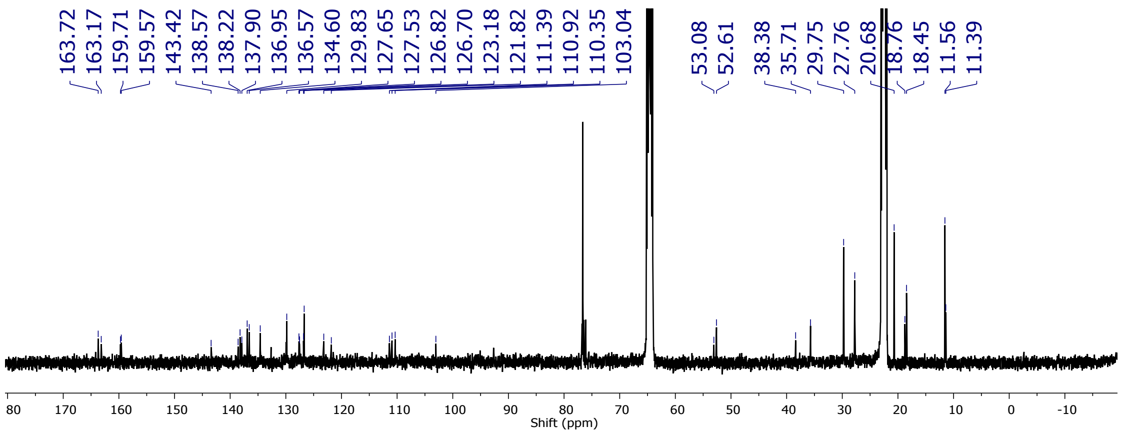

Figure S10. ^{13}C NMR of compound **VC10** (100 MHz, THF-d_8).

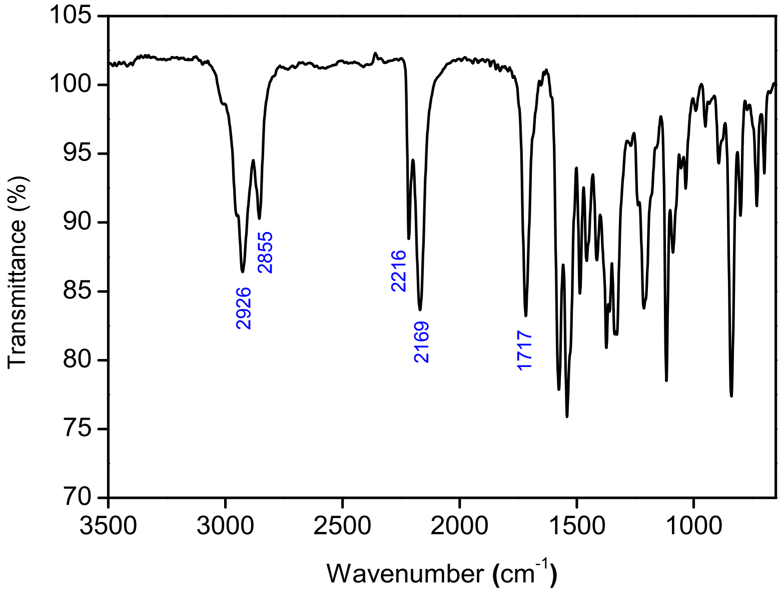

Figure S11. FT-IR spectrum of compound **VC10** (ATR).

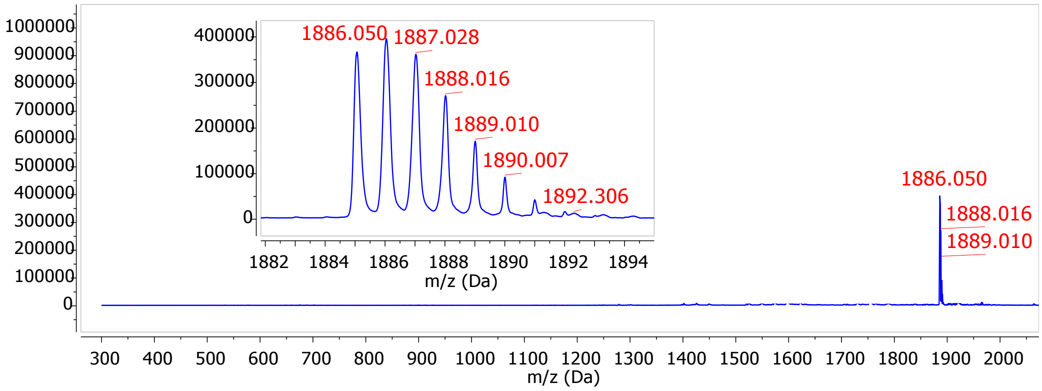

Figure S12. MALDI-TOF MS spectrum of compound **VC10**.

3. Thermogravimetric analysis (TGA) of VC10.

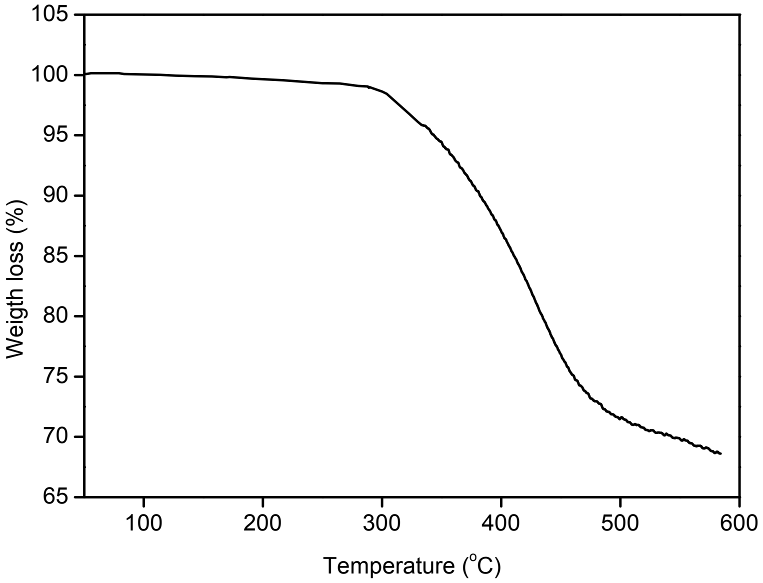

Figure S13. Thermogravimetric analysis of **VC10** (T^a_d= 424 °C, calculated from 1st derivative).

4. Electrochemical studies.

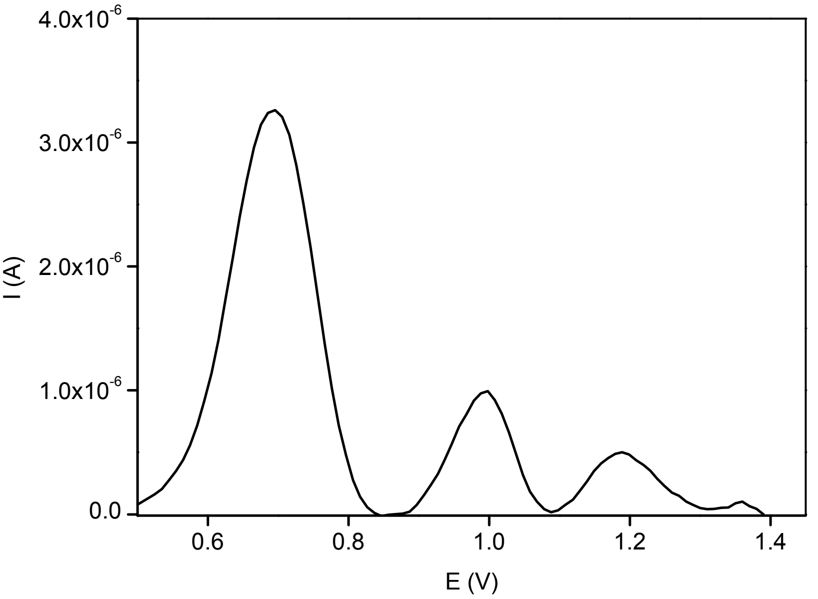

Figure S14. Anodic window (OSVW voltammetry) of **VC10**.

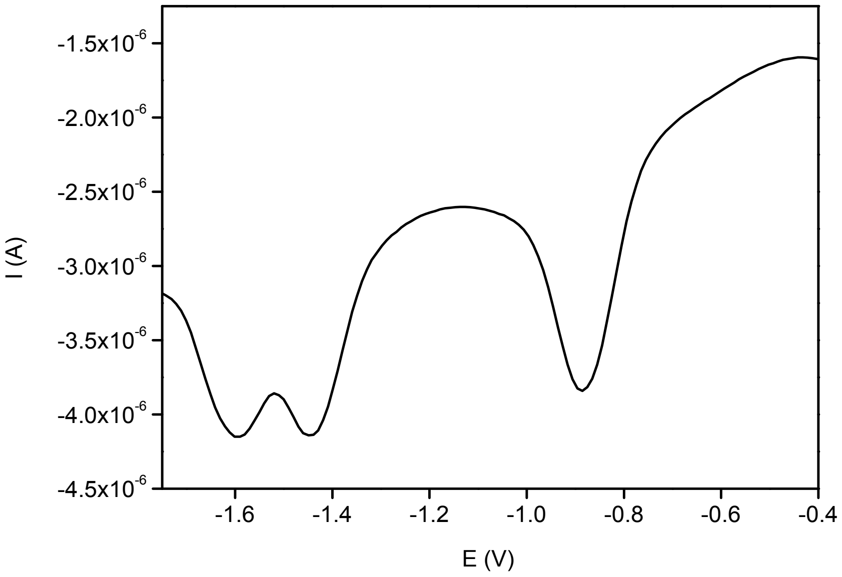

Figure S15. Cathodic window (OSVW voltammetry) of **VC10**.

5. Theoretical Calculations

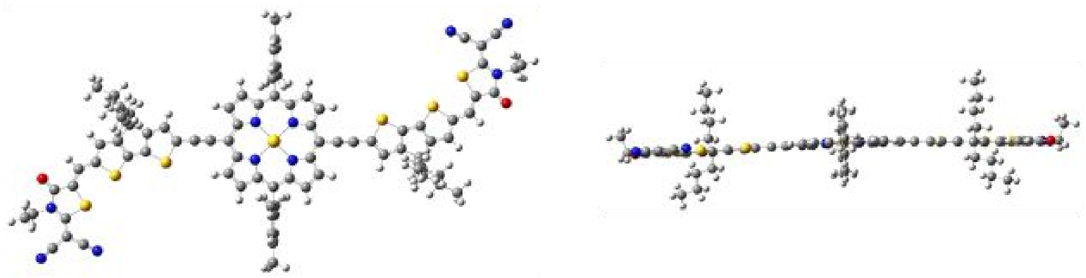

Figure S16. Theoretical optimized geometry of compound **VC10**.

6. Photovoltaic Studies

a)

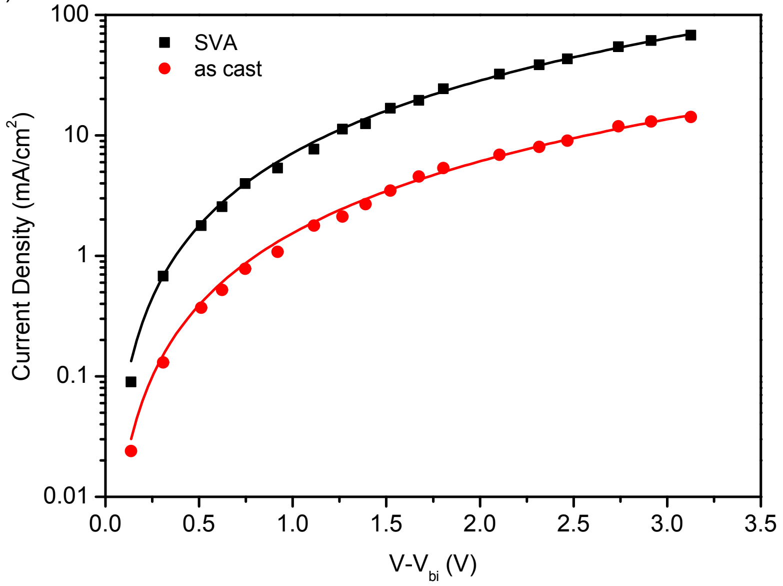

b)

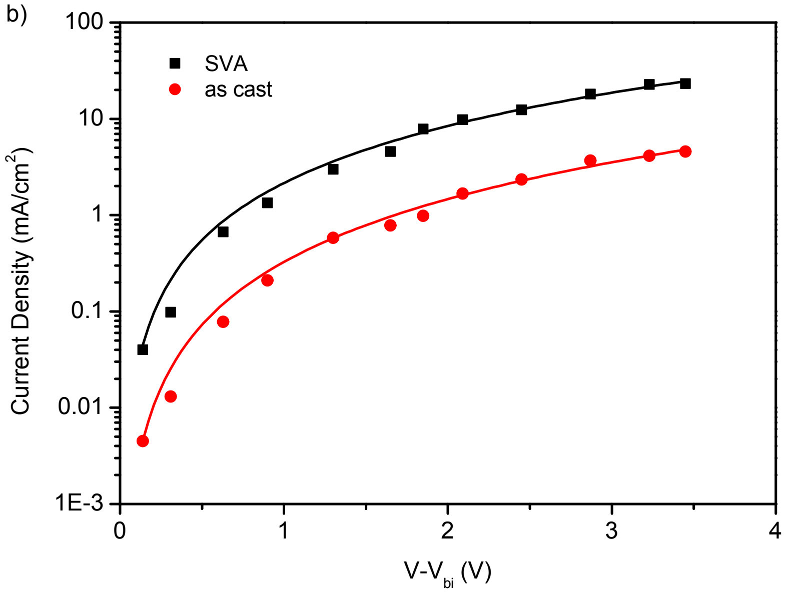

Figure S17. Dark J-V characteristics of (a) hole-only and (b) electron-only devices using as-cast and SVA-treated PTB7-Th:**VC10** films.

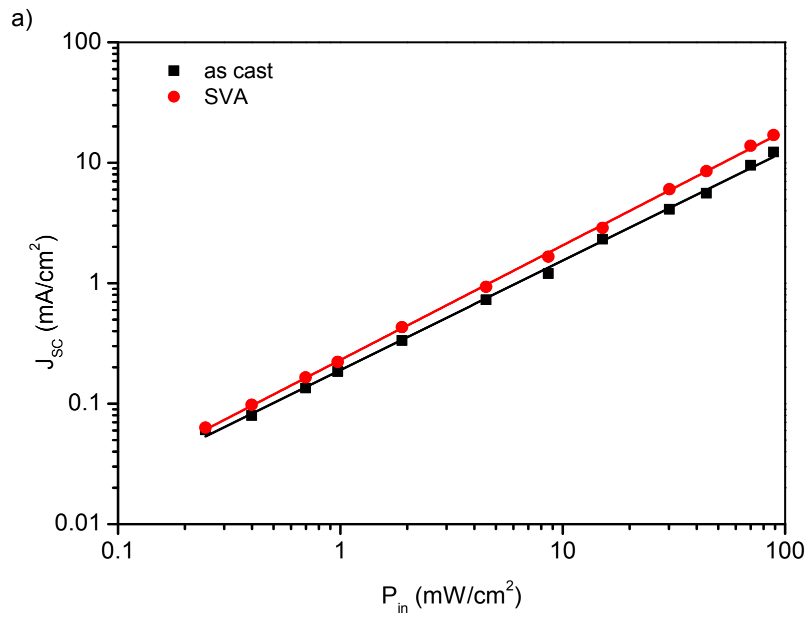

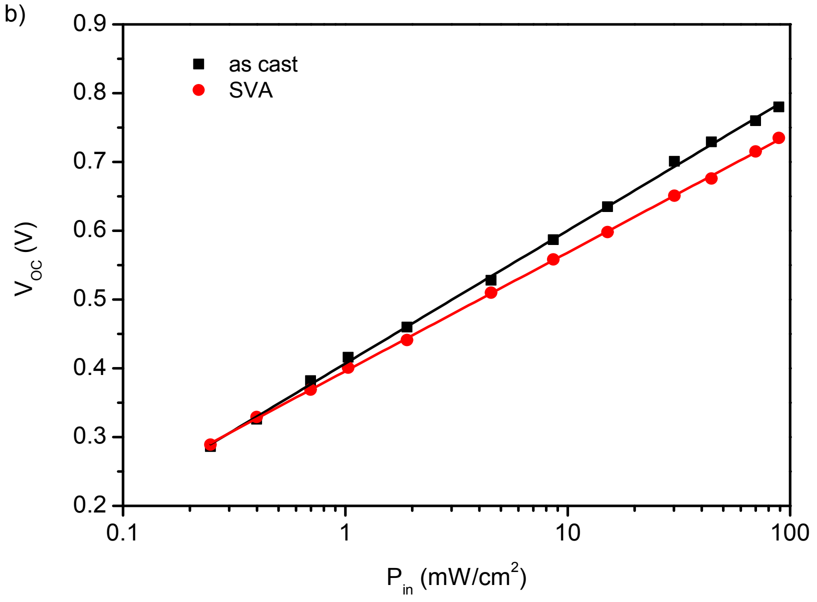

Figure S18. Variation in (a) J_{SC} and (b) V_{OC} for the OSCs based on as-cast and SVA-treated PTB7-Th:**VC10** films.

Table S1a. Photovoltaic parameters of the PTB7-Th:**VC10** based PSCs for different weight ratio of PTB7-Th and **VC10**.

D:A weight ratio	J_{SC} (mA/cm^2)	V_{OC} (V)	FF	PCE (%)
1:0.4	11.02	0.85	0.49	4.59
1:0.8	12.78	0.87	0.51	5.67
1:1.2	13.30	0.87	0.53	6.13
1:1.3	12.74	0.86	0.52	5.70

Table S1b. Photovoltaic parameters of the PSCs based on PTB7-Th:**VC10** (1:1.2) active layer subjected to SVA treatment for different times.

SVA exposure time	J_{SC} (mA/cm^2)	V_{OC} (V)	FF	PCE (%)
10 s	15.98	0.84	0.57	7.65
30 s	16.71	0.83	0.60	8.32
40 s	17.67	0.83	0.63	9.24
50 s	17.01	0.82	0.62	8.69

7. GIWAX

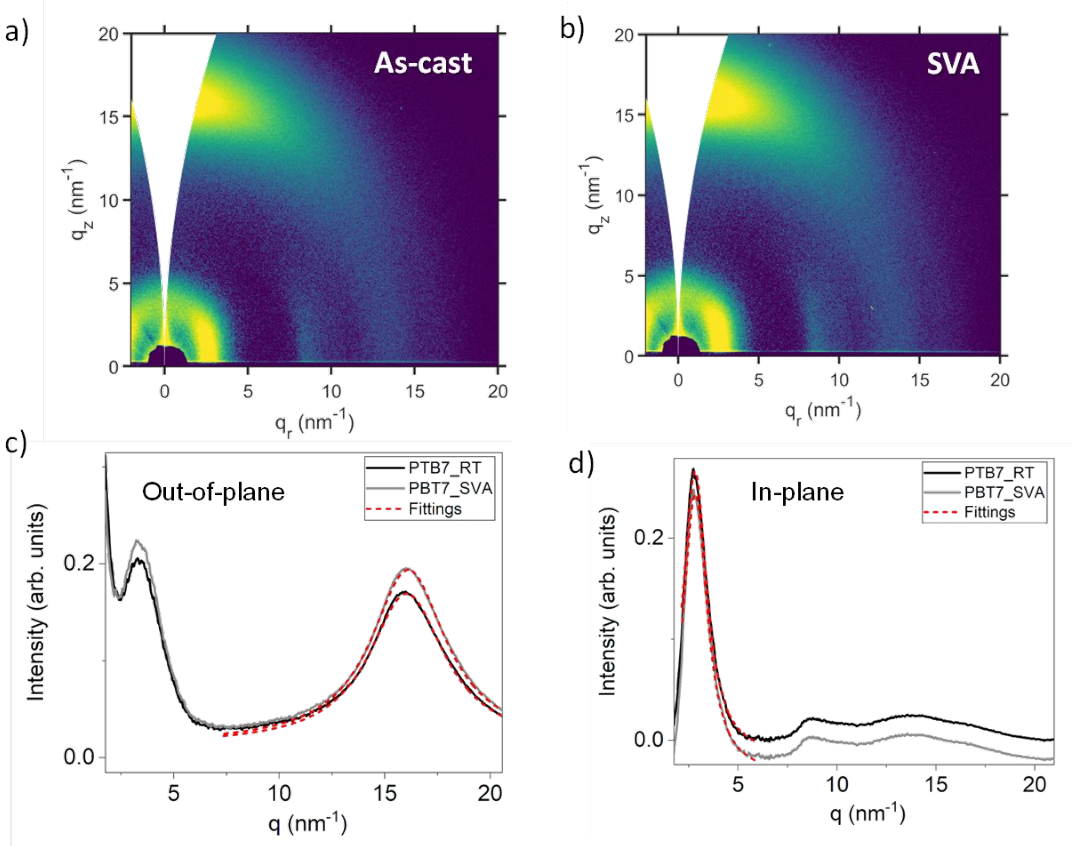

Figure S19. (a-b) GIWAXS patterns from the PTB7 samples as-cast and after SVA procedure. (c-d) Grey and black curves correspond to intensity profiles obtained by azimuthal integrations of the GIWAXS patterns along the q_z axis (Out-of-plane) and along the q_r axis. Red curves correspond to fittings to Lorentzian functions.

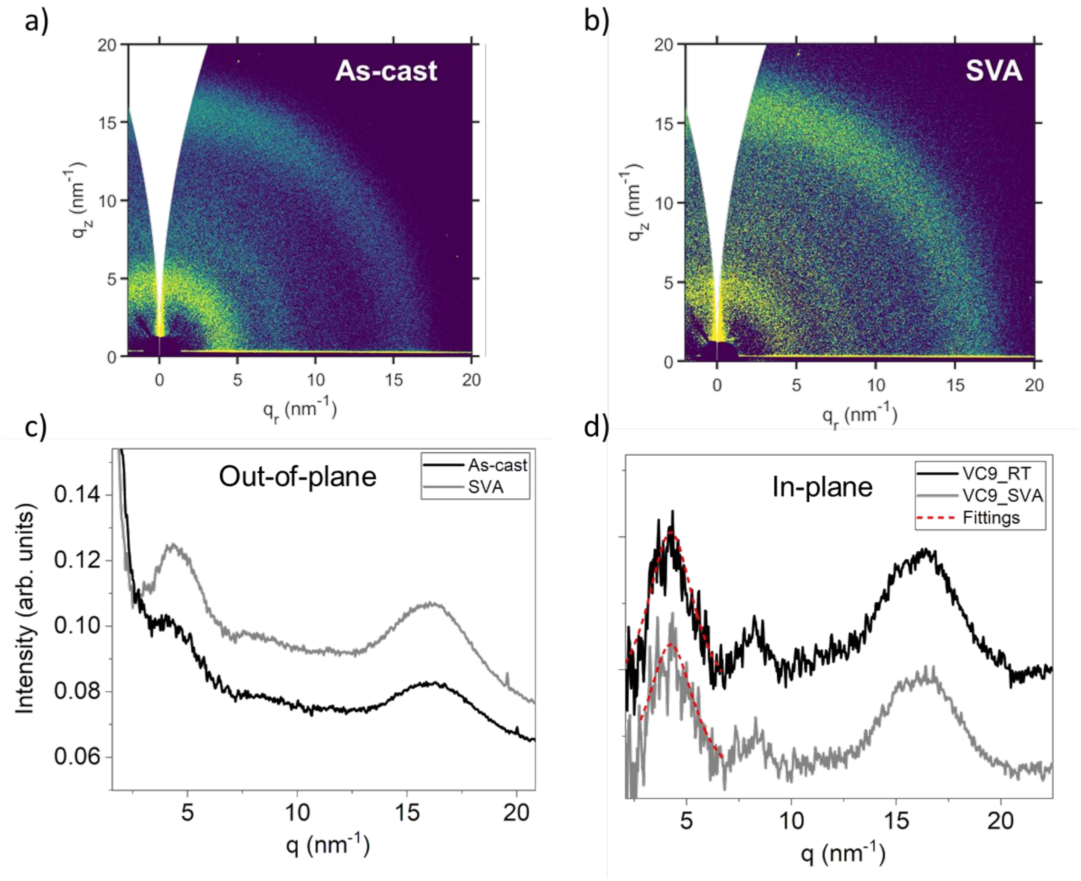

Figure S20. (a-b) GIWAXS patterns from the **VC10** samples as-cast and after SVA procedure. (c-d) (Grey and black curves) Intensity profiles obtained by azimuthal integrations of the GIWAXS patterns along the q_z axis (Out-of-plane) and along the q_r axis (In-plane). Red curves correspond to fittings to Lorentzian functions.

Peak fitting:

All the peaks were fitted using Lorentzian peak profiles. The intensity profiles were corrected by subtracting the intensity scattered by the air. In the case of in-plane peaks, the diffuse intensity near the direct beam was subtracted by modeling the intensity decay with an exponential function (y=A+B*exp(-x/C).

	q (nm^{-1})	FWHM (nm-1)	d (nm)
**PTB7_RT_IP**	2.83	1.44	2.22
**PTB7_SVA_IP**	2.84	1.44	2.21
**VC9_RT_IP**	4.25	2.84	1.48
**VC9_SVA_IP**	4.25	2.83	1.48
**Blend_RT_IP_1peak**	3.04	1.62	2.07
**Blend_RT_IP_2peak**	4.29	3.60	1.47
**Blend_SVA_IP_1peak**	3.02	1.41	2.08
**Blend_SVA_IP_2peak**	4.29	3.66	1.46
**PTB7_RT_OOP**	16.04	4.39	0.392
**PTB7_SVA_OOP**	16.12	4.35	0.390
**Blend_RT_OOP**	17.03	3.27	0.369
**Blend_SVA_OOP**	17.07	3.51	0.368
